# Acaricidal efficacy of orally administered macrocyclic lactones against poultry red mites (*Dermanyssus gallinae*) on chicks and their impacts on mite reproduction and blood-meal digestion

**DOI:** 10.1186/s13071-019-3599-0

**Published:** 2019-07-12

**Authors:** Xiaolin Xu, Chuanwen Wang, Shudong Zhang, Yu Huang, Tingting Pan, Bohan Wang, Baoliang Pan

**Affiliations:** Department of Veterinary Parasitology, College of Veterinary Medicine, China Agricultural University, Hai Dian District, Beijing, 100193 China

**Keywords:** *Dermanyssus gallinae*, Poultry red mite, Oral, Reproduction, Blood-meal digestion, Macrocyclic lactones

## Abstract

**Background:**

The poultry red mite (PRM), *Dermanyssus gallinae*, is one of the most economically deleterious threats to laying-hen industry worldwide. Macrocyclic lactones (MLs) have been widely used in control of mites in mammals, but the effects of MLs on PRMs are not well studied. The main objective of the present study was to systematically evaluate the effects of three MLs, i.e. eprinomectin (EPR), moxidectin (MOX) or ivermectin (IVM), on PRMs fed on chicks following oral administration.

**Methods:**

Chicks in treatment groups were orally administrated with EPR, MOX or IVM at a dose of 5.0 mg/kg bodyweight. Chicks in the control group received the carrier solvent without drug. Chicks in each cage were then infested with 200 starved adult *D. gallinae*. After infestation and feeding for 12 h, engorged mites were collected to evaluate the acaricidal efficacy of the MLs, and its impacts on the reproduction and blood-meal digestion of *D. gallinae*.

**Results:**

MOX, IVM and EPR demonstrated higher acaricidal efficacies post-treatment compared with the control, i.e. 45.60% for MOX, 71.32% for IVM and 100% for EPR on Day 10. MLs did not have significant effects on the blood-meal ingestion of PRMs, but significantly slowed down blood digestion (*P* < 0.0001). The oviposition rate, egg hatching rate and fecundity of PRMs in treatment groups were remarkably reduced. Among the three MLs, EPR exhibited the highest performance against PRMs, with an oviposition rate of 1.04%, fecundity of 0.33 eggs per mite and a zero egg hatching rate in EPR treated groups.

**Conclusions:**

EPR, MOX or IVM administrated orally to chicks increased the mortality of *D. gallinae*, significantly slowed down their blood-meal digestion and significantly reduced their reproductive capability which included the oviposition rate, fecundity and egg hatching rate. The present study highlights the potential of MLs in the control of PRMs.

## Background

*Dermanyssus gallinae*, the poultry red mite (PRM), is a significant threat to laying hens in many parts of the world, including Europe, Japan and China [1–3]. This nocturnal hematophagous ectoparasite has a rapid rate of proliferation with a negative impact on the birds’ health, welfare and productivity, as well as the egg quality, resulting in severe economic loss [1, 4, 5]. In addition, *D. gallinae* is of growing concerns in human health. *Dermanyssus gallinae* infestation is increasingly responsible for human dermatological lesions, namely gamasoidosis, particularly in people living or working in close proximity to poultry [6]. After being bitten by *D. gallinae*, the person may develop erythematous, papular eruptions usually associated with itching. Apart from its potential role in eliciting allergic reactions, *D. gallinae* may also act as a vector of infectious pathogens [2, 7]. *Dermanyssus gallinae* populations have a significant impact on poultry health as this species can serve as a pathogen vector [5, 8, 9], with any individual mite potentially harbouring multiple pathogens [9]. Although this vectorial role for some pathogens remains to be definitively confirmed, their potential to spread disease should not be underestimated [1, 5, 6]. Poultry red mites are very resistant to desiccation and starvation, enabling them to survive up to eight months without feeding on a host [1]; as a consequence, remaining *D. gallinae* can continuously multiply and build up a population over many production cycles, which often makes a one-time treatment of poultry houses against *D. gallinae* challenging. Therefore, there is an urgent need for an effective method of controlling *D. gallinae.*

The traditional methods against *D. gallinae* have relied on a range of acaricides, including carbamates, organophosphates, amidines, pyrethroids, and more recently spinosad, applied to premises and birds as sprays, mists and dusts [10, 11]. There are, however, many limitations of these approaches, including barely achieving acaricidal levels in all the hard-to-reach harboring sites of *D. gallinae* [1], causing stress to the birds, and the risks of residues and pesticide exposure to the workers [12]. In addition to the above, the emergence of resistance to available acaricides is one of the main reasons for the failure of acaricides to manage *D. gallinae* [13–18]. The resistance to organophosphates, pyrethroids, carbamates and dichlorodiphenyltrichloroethane (DDT) has been found in *D. gallinae* populations across the EU [10, 11]. Therefore, there is a long-recognized need for novel methods of reducing or even eliminating the threats arising from *D. gallinae* infestations [1]. There is an urgent requirement for research to uncover more efficient control strategies.

Administering medical treatment *via* the oral route is considered as the preferred method to control red mite populations in the poultry industry. This systemic treatment approach is highly effective and convenient, and can help to overcome the limitations in current methods for *D. gallinae* control because any vital blood-feeding mite stage in a poultry house will inevitably feed on treated poultry [19]. It has been demonstrated that high efficacies (97.8–100%) of a novel acaricide (fluralaner) against PRMs were obtained on commercial poultry farms in France, Germany and Spain when it was orally administrated twice *via* drinking water [20]. As an isoxazoline compound, fluralaner paralyzes and kills mites through binding at a distinct receptor site on γ-aminobutyric acid (GABA)-gated and L-glutamate-gated chloride channels, which are widely expressed in the nervous systems of acarids and insects [21]. The mechanism of action of fluralaner is similar to that of macrocyclic lactones [22, 23].

Macrocyclic lactones (MLs), mainly including two types of compounds, avermectins (abamectin, ivermectin, doramectin, eprinomectin, selamctin) and milbemycins (moxidectin, milbemycin oxime), are broad-spectrum antiparasitic drugs, which have been widely used to control endo- and ecto-parasites in livestock and pets as well as humans. MLs show very high potency against various mites in livestock and pets, such as *Sarcoptes*, *Psoroptes*, *Chorioptes* and *Otodectes*, at a low dosage of 0.2 mg/kg (for subcutaneous administration) or 0.5 mg/kg (for topical administration) [24–29]. Interestingly, only a few studies have been conducted on the efficacy of MLs against the PRMs, which showed that the efficacy of MLs against PRMs was quite low in comparison to those against mites in mammals. For example, it was found that the intra-abdominal injection of birds with ivermectin at 0.6 mg/kg was insufficient to control *D. gallinae* and efficacious concentrations were from 1.8 to 5.4 mg/kg [30, 31]. In addition, after the initial treatment, ivermectin and selamectin were most efficacious against *D. gallinae* on Day 16, and moxidectin was most effective in control of mites on Day 24 [32]. Compared with intraperitoneal injection or subcutaneous administration, oral administration is more convenient to birds (particularly for a large number of birds on poultry farms). However, no literature on the efficacy of MLs against PRMs following oral administration is available. The effects of MLs on the PRMs are not well established, such as the effects on the PRMs’ oviposition rate, fecundity and the hatching rate of eggs, and blood ingestion and blood-meal digestion rate.

Hence, the objective of the present study was to study the effects of oral administration of three MLs on PRMs in chicks, i.e. eprinomectin (EPR), moxidectin (MOX) and ivermectin (IVM). The evaluation parameters of effects included the acaricidal efficacy, the effects on the reproduction capability and blood-meal digestion of PRMs.

## Methods

### Drugs

IVM (purity > 93.73%) and EPR (purity > 95.74%) were purchased from Hebei Veyong Animal Pharmaceutical Co., Ltd. (Shijiazhuang, China). MOX (purity > 96.40%) was purchased from Zhejiang HISUN Pharmaceutical Co., Ltd. (Taizhou, China).

### Animals

All chicks used in the study were hatched under laboratory conditions and kept in metal cages and plastic storage boxes, and received feed and water *ad libitum*. The storage boxes with chicks were placed in an artificial climate incubator (RXZ-500B-LED, Ningbo Jiangnan Instrument Factory, Ningbo, China), with 30 ± 2 °C and 75 ± 2% relative humidity (RH), and light was provided by the incubator with a light intensity of approximately 3600 lux with a 12:12 h (L:D) photoperiod [33].

### Grouping and treatment

When the chicks were 6 weeks old, they were assigned randomly to treatment or control groups. Each group included three replicates (i.e. I, II and III). There were three chicks in a replicate. All chicks were clinically examined and weighed 1 day before the administration (Day 0).

EPR, MOX or IVM was fully dissolved in an ethanol/PBS/Tween 80 mixture in a 40:58:2 ratio (v/v) to formulate a 2 mg/ml solution [34]. On Day 0, chicks in the treatment groups were orally administered of EPR, MOX or IVM at a dose of 5 mg/kg body weight by gavage, while chicks in the negative control groups were given carrier solvent without drug. The dose was chosen based on preliminary trials. Approximately 1, 6 and 24 h after the oral administration, chicks were observed for possible adverse reactions. The chicks’ general health was monitored daily to ensure safety and health during the study.

### Mite infestation and collection

*Dermanyssus gallinae* were taken from a laboratory culture, which was kept in a rearing system with chicks under laboratory conditions [33]. All adult female mites used for infestation (challenge) were starved for 3 days prior to infestation to increase the feeding rate [19]. Before the challenge, ten trap tubes were placed in a new metal cage at the aggregation sites of mites at the bottom of the cage to trap challenged mites [33]. After treatments (on Day 0), the chicks were put back into the metal cage and were immediately challenged with 200 starved adult female mites. For the challenge, two trap tubes containing 200 starved female mites were placed at the bottom of the cage and were opened to let the mites move out of the tubes and infest the birds. Then, the metal cages were put in plastic storage boxes and the rearing systems were kept in an artificial climate incubator without lighting for 12 h to enable the mites to feed on the chicks. Thereafter, all visible engorged adult mites including live or dead mites were collected from all trap tubes, the metal cage and plastic storage box after removal of the chicks.

Three independent studies were conducted: (i) the acaricidal efficacy of EPR, MOX or IVM against PRM; (ii) the impacts of MLs on the reproduction of *D. gallinae*; and (iii) the impacts of MLs on blood-meal digestion of *D. gallinae*. In each study, 200 starved adult female mites were challenged to each cage. Twelve hours after the challenge, all visibly engorged mites were collected and incubated under 30 ± 2 °C, 75 ± 2% RH, and a 12:12 h L:D photoperiod (in incubator) for further evaluation as described below.

#### Study 1: acaricidal efficacy

The number of engorged dead mites was recorded at 12 h post-treatments, and the engorged live mites were kept in 96-well round bottomed ELISA plates and were observed daily under a stereomicroscope (SteREO-Discovery.V12; Carl Zeiss, Jena, Germany) for 10 days. The apertures of the 96-well round bottomed ELISA plates were closed by plastic lids in a way that allowed air exchange but prevented mites from escaping. The mites were determined dead if neither light nor mechanical stimulation elicited an active movement of the legs; the numbers of both dead and live mites were recorded daily.

Efficacy against adult mites was calculated at each assessment day. The following formula [19] was used

$$ {\text{Efficacy}} = \frac{{{\text{Mt}} - {\text{Mc}}}}{{1 - {\text{Mc}}}} \times 100{{\% }} $$

where Mt and Mc are the adult mite mortality rate in the treatment and control group, respectively.

#### Study 2: mite reproduction

The oviposition of female mites and the hatchability of eggs were evaluated following a previously described method [35]. After collection as mentioned above, the mites in 96-well round bottomed ELISA plates were incubated for 1 week and observed using a stereomicroscope. The number of mites laying eggs and the total number of eggs were counted to determine their oviposition rates and mite fecundity. In addition, the numbers of hatched larvae were also recorded by using a stereomicroscope.

The effects of compounds on the reproductive capacity of *D. gallinae* were evaluated by comparing the number of mites laying eggs, eggs and larvae between the treatment and the control group. The reproduction parameters were calculated as below [36]:

$$ {\text{Oviposition rate}}\, = \,\frac{{{\text{No}}.\,   {\text{of mites laying eggs}}}}{{{\text{No}}.\,   {\text{of mites}}}}\, \times \,100{{\% }} $$

$$ {\text{Fecundity}} = \frac{{{\text{No}}.\,   {\text{of eggs }}}}{{{\text{No}}.\,   {\text{of mites laying eggs}}}} \times 100\% $$

$$ {\text{Hatchability}}\, = \,\frac{{{\text{No}}.\,   {\text{of larvae}}}}{{{\text{No}}.\,   {\text{of eggs}}}}\, \times \,100{{\% }} $$

#### Study 3: blood-meal digestion

The blood-meal digestion of *D. gallinae* was assessed by observing changes in appearance after treatments under a stereomicroscope and weighing mites at different periods of digestion. The adult mites starved for 3 days were weighed, and the average body weight was calculated before infestation. All live engorged mites were weighed when mites were collected, and then were transferred into 100-ml centrifuge tubes. These mites were kept in incubator and observed for 5 days. On Day 1, 3 and 5, survived mites were removed from the centrifuge tube, observed and weighed, while the dead mites were excluded. After observation, live mites were transferred to new 100-ml centrifuge tubes and placed back in the incubator.

The average weight of each mite in each count point was calculated after weighing. The effects of treatment on adult mites’ ingestion and blood digestion were evaluated by calculating the ingestion rate and digestion rate [37]:

$$ {\text{Ingestion rate}}\, = \,\frac{\text{Average weight gain after feeding}}{\text{Average body weight before feeding}}\, \times \,100{{\% }} $$

$$ {\text{Digestion rate}}\, = \,\frac{{{\text{Average weight after feeding}} - {\text{Average weight after digesting period}}}}{\text{Average weight gain after feeding}}\, \times \,100{{\% }} $$

### Data analysis

We used three replicates for each treatment. All data were conducted and statistically analyzed using one-way ANOVA in SPSS v.20.0 software (SPSS Inc., Chicago, IL, USA). *P*-values less than 0.05 (*P* < 0.05) were considered to be statistically significant.

## Results

### Acaricidal efficacy

**Fig. 1:**
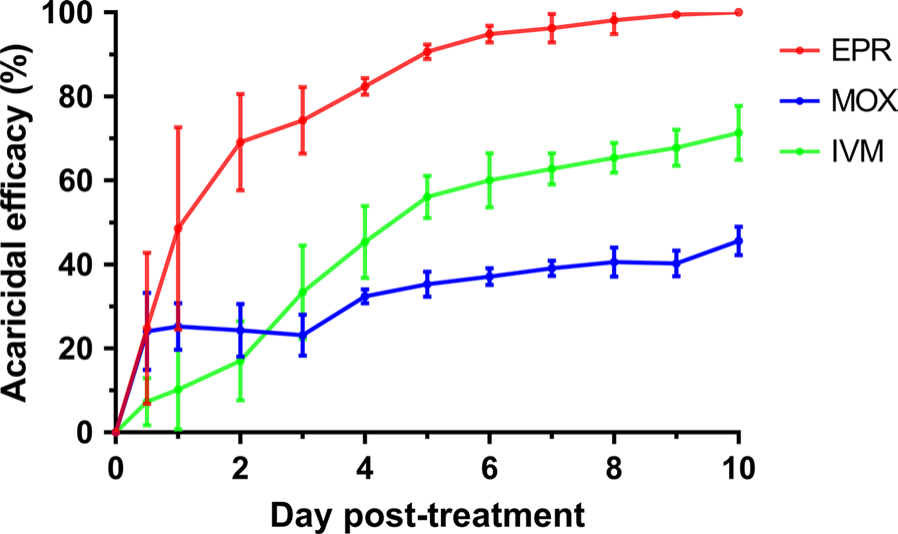
Acaricidal efficacy (%) of *D. gallinae* after blood-feeding on EPR, MOX or IVM-treated chicks. The acaricidal efficacy of mite were assessed every 24 h for 10 days after mite collection. All values are shown as the mean ± SD from three independent experiments. *Abbreviations*: EPR, eprinomectin; MOX, moxidectin; IVM, ivermectin

Figure 1 shows the acaricidal efficacy of three MLs against adult PMs. The acaricidal efficacy in the EPR-treated group gradually increased, from 48.57% on Day 1 to 90.64% on Day 5, achieving 100% on Day 10. For the MOX-treated group, the acaricidal efficacy reached 45.60% on Day 10. The acaricidal efficacy for the IVM-treated group was 71.32% on Day 10. The acaricidal efficacy in the MOX-treated group and the IVM-treated group were both significantly lower than that of the EPR-treated group on Day 2 (*F*_(2, 6)_ = 0.24, *P* = 0.001), Day 3 (*F*_(2, 6)_ = 0.22, *P* = 0.001), Day 4 (*F*_(2, 6)_ = 0.20, *P*  < 0.0001), Day 5 (*F*_(2, 6)_ = 0.23, *P*  < 0.0001), Day 6 (*F*_(2, 6)_ = 0.25, *P*  < 0.0001), Day 7 (*F*_(2, 6)_ = 0.25, *P*  < 0.0001), Day 8 (*F*_(2, 6)_ = 0.25, *P*  < 0.0001), Day 9 (*F*_(2, 6)_ = 0.26, *P*  < 0.0001) and Day 10 (*F*_(2, 6)_ = 0.22, *P*  < 0.0001).

### Mite reproduction parameters

The oviposition rate, fecundity and egg hatching rate of *D. gallinae* are shown in Table 1. The oviposition rates, fecundities and egg hatching rates of mites in all three treatment groups were obviously lower than those in control group. Compared with the values in the control group, the oviposition rates of mites fed on EPR, MOX and IVM-treated chicks were significantly reduced by 94.77, 92.7 and 87.95%, respectively. Similarly, the average numbers of eggs per mite produced in EPR, MOX and IVM groups were 0.33, 1.00 and 0.91, respectively, while the average number of eggs for the control group was 3.61; these differences between the control group and the three treatment groups were significant (*F*_(3, 8)_ = 6.41, *P* = 0.002). No eggs hatched in the EPR-treated group (i.e. zero hatching rate) and IVM significantly decreased the hatching rate (26.26%); values in both groups were significantly lower (*F*_(2, 6)_ = 0.78, *P* < 0.0001) than that of the control group (98.69%). Although MOX remarkably decreased the hatching rate (60%), no significant difference existed compared with control group due to the high variety among three replicates of MOX treated groups (Table 1).

**Table 1:** Oviposition rate (%), fecundity (%) and egg hatching rates (%) of adult female mites

Reproduction parameters	Repetitions	Control	EPR	MOX	IVM
Oviposition rate (%)	I	95.95	3.13	0.00	0.00
	II	97.73	0.00	4.92	15.25
	III	93.75	0.00	4.41	8.33
Mean (%)		95.81 ± 1.99^c^	1.04 ± 1.80^a^	3.11 ± 2.71^a^	7.86 ± 7.64^b^
Fecundity (%)	I	3.82	1.00	0.00	0.00
	II	3.88	0.00	1.33	1.22
	III	3.13	0.00	1.67	1.50
Mean (%)		3.61 ± 0.42^b^	0.33 ± 0.58^a^	1.00 ± 0.88^a^	0.91 ± 0.80^a^
Hatching rate (%) (A/B)	I	97.79 (265/271)	0 (0/1)	0 (0/0)	0 (0/0)
	II	100 (167/167)	0 (0/0)	100 (4/4)	45.45 (5/11)
	III	98.30 (231/235)	0 (0/0)	80.00 (4/5)	33.33 (2/6)
Mean (%)		98.69 ± 1.16^c^	0 ± 0^a^	60.00 ± 52.92^bc^	26.26 ± 23.54^ab^

I, II and III represent three replicates of each group

^a, b, c, d^Values within the same row followed by different letters are significantly different

(A/B): A refers to the number of eggs hatched into larvae, B refers to the number of eggs laid by all mites

The data of mean (%) are expressed as the mean ± SD

*Abbreviations*: EPR, eprinomectin; MOX, moxidectin; IVM, ivermectin

### Blood-meal digestion

**Fig. 2:**
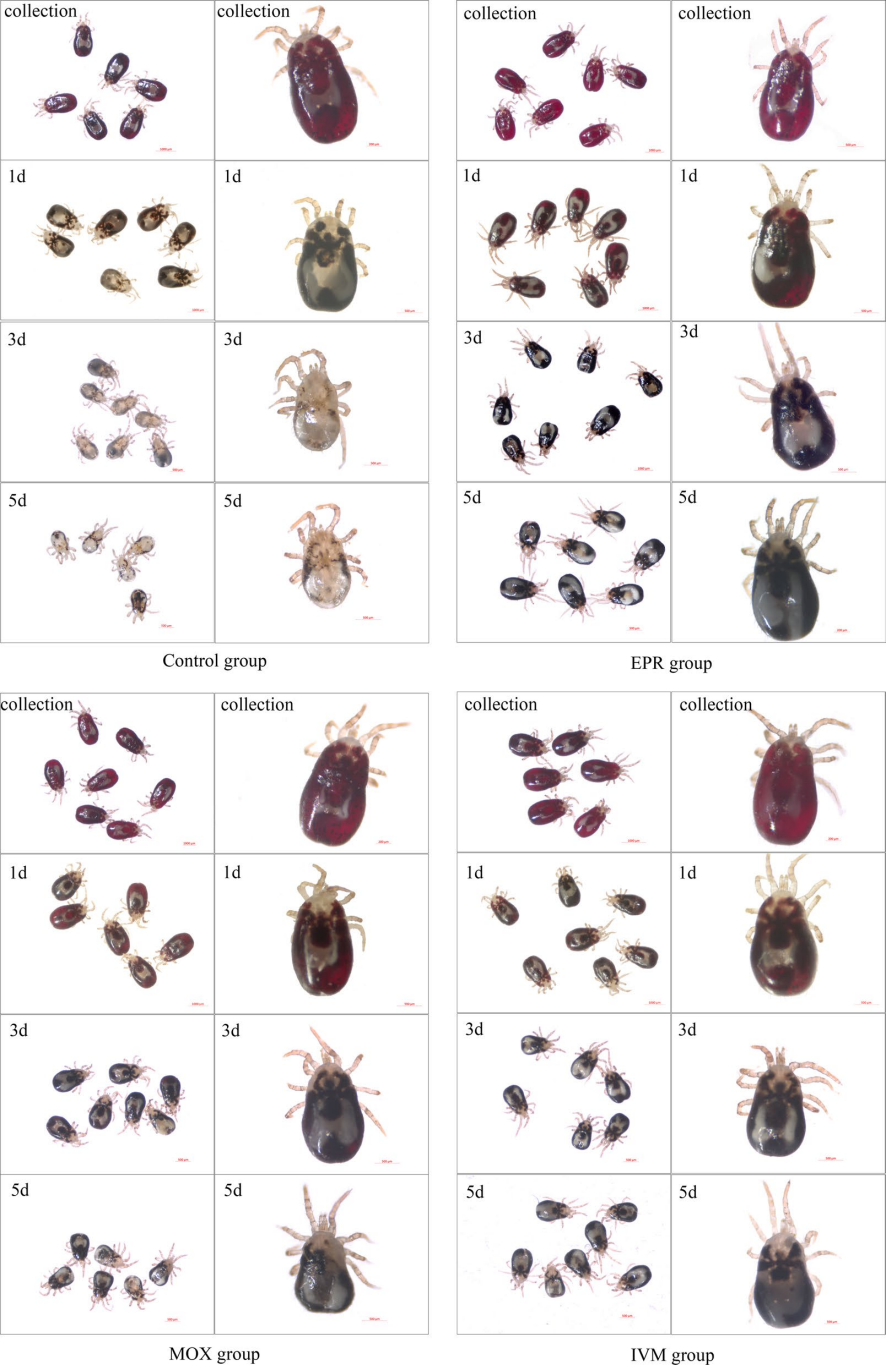
Effect of EPR, MOX or IVM on mite appearance at digestion periods. Engorged mites in control or treatment groups were collected and observed by stereomicroscope on 12 h (collection) after infestation. Several mites were randomly selected from the surviving mites and observed on Day 1 (1d), Day 3 (3d) and Day 5 (5d) after collection, and the appearance and blood changes of one mite were observed carefully. *Abbreviations*: EPR, eprinomectin; MOX, moxidectin; IVM, ivermectin

**Fig. 3:**
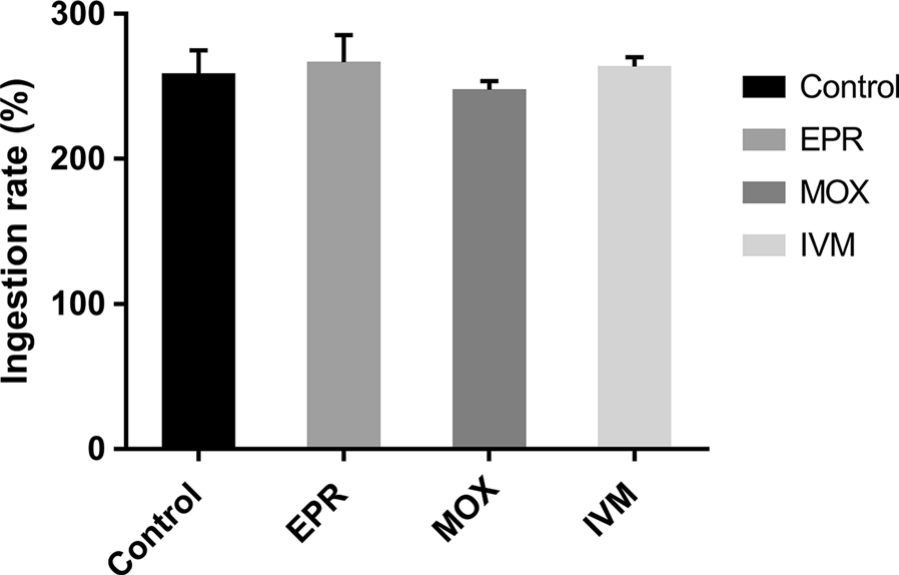
Effect of treatment (EPR, MOX or IVM) on blood ingestion by adult mites collected at 12 h. Engorged mites in control or treatment groups were collected, weighed and calculated at 12 h after infestation. The data are expressed as the mean ± SD. *Abbreviations*: EPR, eprinomectin; MOX, moxidectin; IVM, ivermectin

We assessed the blood-meal digestion of *D. gallinae* by observing changes in appearance and weighing mites at different periods. As shown in Fig. 2, there was no obvious difference in mite appearance when they were collected from treatment or control groups, e.g. the mites were red and full of blood. The ingestion rates of mite are shown in Fig. 3, that there were no significant differences between treatment groups and control group, indicating that the orally administered MLs did not significantly affect the blood ingestion of *D. gallinae*. However, as shown in Table 2, blood-meal digestions of mites in the treatment groups were significantly lower than those in the control group on Day 1 (*F*_(3, 8)_ = 0.04, *P* < 0.0001), Day 3 (*F*_(3, 8)_ = 0.21, *P* < 0.0001) and Day 5 (*F*_(3, 8)_ = 0.11, *P* < 0.0001) after mite collection. On Day 3 after mite collection, the blood in mites in control group was almost completely digested and the digestion rate reached 94.41%, while the digestion rates were 36.58, 68.74 and 42.22% in the EPR-, MOX- and IVM-treated groups, respectively (Table 2). On Day 5 after collection, the digestion rates were 60.74, 75.03 and 56.23% in the EPR-, MOX- and IVM-treated groups, respectively (Table 2), which were significantly lower than that (99.36%) in control group (*F*_(3, 8)_ = 0.11, *P* < 0.0001).

**Table 2:** Effects of treatment (EPR, MOX or IVM) on blood-meal digestion of adult mites

Days after mite collection	Digestion rate (%)			
	Control	EPR	MOX	IVM
1	47.91 ± 1.19^a^	21.25 ± 0.78^b^	33.37 ± 3.81^c^	26.04 ± 0.64^d^
3	94.41 ± 1.60^a^	36.58 ± 2.59^b^	68.74 ± 2.86^c^	42.22 ± 1.93^d^
5	99.36 ± 0.75^a^	60.74 ± 1.87^b^	75.03 ± 1.48^c^	56.23 ± 0.23^d^

^a, b, c, d^Values within the same row followed by different letters are significantly different

The data are expressed as the mean ± SD

*Abbreviations*: EPR, eprinomectin; MOX, moxidectin; IVM, ivermectin

The observed appearance of mites was in agreement with the result of weighing. As shown in Fig. 3, it was obvious that there was still a large quantity of dark red blood in the caeca of survived adult mites in treatment groups, while mites in control group had a little residual blood at the edge of caeca on Day 1 after mite collection. It illustrated the slower blood digestion of mites in treatment groups than that in control group. On Days 3 and 5 after mite collection, there was still blood that had become black at the edge of the caeca in treatment groups, while there was almost no blood residue and the mite was nearly transparent in the control group, indicating that the blood meal was digested completely. All these results confirmed that blood-meal digestion of *D. gallinae* was interrupted or slowed down by EPR, MOX or IVM.

### Health observations

There were no adverse responses in all birds of any study during the whole period that was considered related to treatment with EPR, MOX or IVM.

## Discussion

The present study was carried out to systematically evaluate the effects of EPR, MOX and IVM on *D. gallinae* in chicks following oral administration at a dose of 5 mg/kg. To the best of our knowledge, this is the first study to assess the acaricidal efficacy of MLs, and the effects on reproduction and blood-meal digestion of *D. gallinae* after oral treatments. The results showed a single oral administration of MOX, IVM or EPR could lead to a significant increase in acaricidal efficacy on Day 10 post-treatment compared with control, i.e. 45.60% for MOX, 71.32% for IVM and 100% for EPR (Fig. 1). Although MLs showed high efficacy against PRMs in the present study, the dose was quite high in comparison to those used in the control of mammal mites. In mammals, MLs exhibit high efficacy against mites at very low dosage, i.e. generally at 0.2 mg/kg for subcutaneous administration or 0.5 mg/kg for topical delivery, which is 25 and 10 times lower, respectively, than the present dose. The exact reasons for this disparity are still unclear; however, two possible causes may relate to this phenomenon. One cause is the difference of pharmacokinetics of MLs in birds and mammals. It has been demonstrated that a fairly low IVM concentration in plasma was obtained when it was orally administrated to laying hens [38, 39]. The other possible reason is the different sensitivity of blood-sucking PRMs and mammals’ mites to MLs. MLs show high potency against various types of pests at a very low dosage (for example 0.2 mg/kg), e.g. lice, larvae of the Hypodermatidae, Gasterophilidae and Oestridae, mites in mammals, but lower efficacy against some blood-feeding pests. It has been confirmed that *Anopheles albimanus* is nearly impervious to the effects of IVM [40]. Bed bugs exhibited low sensitivity to MOX, and MOX could cause various sublethal effects on bed bugs [37]. However, the exact reasons for the low potency of MLs against PRMs need to be examined by further studies.

To our knowledge, the effects of MLs on blood-meal ingestion, blood digestion and reproduction were observed for the first time in the present study. Interestingly, MLs did not show any negative effects on the blood-meal ingestion of *D. gallinae* (Fig. 3). Two reasons may relate to this phenomenon, namely delayed action of MLs against pests (generally taking effect a long period of time after ingestion) and the short blood-feeding period of PRMs (0.5–1.5 hours) [1]. Similar results have been obtained in bed bugs [37]. Compared with PRMs in the control group, the egg hatching rate, the oviposition rate and the fecundity of PRMs fed on chicks treated with MLs were significantly reduced (*P* < 0.05), with the egg hatching rate in the MOX group being the only exception (Table 1). The blood-meal digestion rates of *D. gallinae* post-treatment were significantly lower than those in the control group (Table 2). It has been demonstrated that egg maturation in various blood-feeding insects (e.g. mosquitos, ticks) depends on the nutrients from blood meals [41, 42]. Furthermore, a previous study demonstrated that the engorgement level of *D. gallinae* had a positive relationship with the reproductive capability [33]. Hence, in the present study, the low blood-meal digestion rate may have caused the low oviposition rate and low fecundity of *D. gallinae*. To our surprise, the egg hatching rate also decreased. It is not clear whether MLs directly interfered the development of embryo in eggs or MLs disturb the transfer of nutrients to eggs. What can be speculated is that inhibitions of MLs on blood-meal digestion and reproduction of *D. gallinae* can result in slowing the proliferation of the *D. gallinae* population. Our findings are consistent with a previous study where the amount of IVM in the blood of treated cattle was sufficient to reduce blood-meal digestion and caused significant mortality and a reproductive decrease of *Anopheles arabiensis* [36]. It has been confirmed that bed bugs that ingested MOX suffer from a decreased digestion rate; in addition, MOX treatment caused sterility of the surviving female bed bugs and significantly reduced their fecundity [37]. Furthermore, a previous study found a great adverse impact on the fertility of female ticks recovered from calves treated with a single dose of 1 mg/kg of EPR [43].

An interesting issue is the different performance of the three MLs against PRMs. Generally, EPR exhibited a higher efficacy and negative effects on the egg hatching rate, the oviposition rate and the fecundity of PRMs than MOX and IVM (Fig. 1, Table 1). The results indicate the EPR has a higher potency against PRMs than IVM and MOX. Similar results have been observed in cattle, whereby it was demonstrated that EPR was more lethal to *Anopheles arabiensis* than IVM and MOX [44].

The present study demonstrates that a high dose of MLs has high acaricidal efficacy against PRMs and has serious negative effects on their reproductive capability and blood digestion, indicating the potential of MLs in control of PRMs. However, several issues need to be addressed before they are applied in poultry farms. These issues include the pharmacokinetics of MLs in laying hens, the efficacy under optional administration programs (for example multiple administrations) and the residue of MLs in eggs.

## Conclusions

To our knowledge, the present study is the first to systematically assess the effects of three MLs on *D. gallinae* on chicks following oral administration. It demonstrates that EPR, MOX or IVM treatment increased the mortality of *D. gallinae* fed on treated chicks, and significantly reduced their reproductive capability, which included the oviposition rate, fecundity and the egg hatching rate. Furthermore, these treatments significantly slowed down the blood-meal digestion of survived mites. Among the three drugs, EPR showed the highest potency on *D. gallinae*. Thus, oral administration of MLs can be potential control method of *D. gallinae* in poultry houses.

## Data Availability

All data generated or analyzed during this study are included in this published article.

## Abbreviations

PRM: poultry red mite
MLs: macrocyclic lactones
EPR: eprinomectin
MOX: moxidectin
IVM: ivermectin
RH: relative humidity
12:12 L:D: 12 h light:12 h darkness photoperiod
Mt: adult mite mortality rate in treatment groups
Mc: adult mite mortality rate in control group
